# Tea consumption is associated with decreased risk of oral cancer: A comprehensive and dose-response meta-analysis based on 14 case–control studies (MOOSE compliant)

**DOI:** 10.1097/MD.0000000000013611

**Published:** 2018-12-21

**Authors:** Hao Zhou, Weiwei Wu, Fengqin Wang, Huizhong Qi, Zhigang Cheng

**Affiliations:** a Department of Oral and Maxillofacial Surgery, The Central Hospital of Wuhan, Tongji Medical College, Huazhong University of Science and Technology, Wuhan; b Jingchu University of Technology, Jingmen, Hubei Province, P. R. China.

**Keywords:** dose-response, meta-analysis, oral cancer, tea

## Abstract

The associations of tea consumption with risk of oral cancer remain not clear. The present meta-analysis aims to clarify the real relationship between tea intake and the risk of oral cancer and quantifies the potential dose-response relationship between them.

A Web search was performed within Pubmed, Embase, and Web of Science databases to identify potential studies that evaluated the relationship between tea consumption and the risk of oral cancer on Mar 21th, 2018 without language restriction. The pooled odds ratios (ORs) with 95% confidence intervals (CIs) were extracted and combined to evaluate the strength of associations. Dose-response analysis was performed to quantitate the relationship between tea intake and risk of oral cancer.

Total 14 articles were included in the final analysis. The pooled OR for evaluating the risk of oral cancer and tea intake was 0.700 (95% CI = 0.609–0.805, *P* <.001). The linearity model of dose-response analysis indicated that with increased 1 cup daily, the risk of oral cancer decreased by 6.2% degree (OR = 0.938, 95% CI = 0.922–0.955, *P* <.001). Subgroup analysis indicated an inverse association between tea intake and the risk of oral cancer except subgroup analysis of black tea and American people.

These results suggest tea intake provides protection against oral cancer carcinogenesis. Additionally, more large-scale pooling and high-quality studies are necessary for detecting the precise relationship between tea intake and oral cancer risk in the future.

## 1 Introduction

Oral cancer (International Classification of Diseases-10: C01–C06), comprises cancer derived from tongue, buccal mucosa, upper or lower gingival, floor of mouth, and hard palate, represents the eighth most frequent cancer in the world.^[1]^ In the United States, oral cancer will account for about 34000 new cases and 7000 deaths in 2018.^[2]^ Although given the fact that new therapy strategy has been improved in recent decades, the 5-year survival rate of oral cancer still remained at 50%.^[3]^ Thus, primary prevention is important to decrease the morbidity and mortality of oral cancer.

Tea, the second popular beverage worldwide except for water, has attracted much attention for its potential cancer preventive effect for many years. The most active molecular for anti-cancer of tea consumption is tea polyphenol, which reported to be associated with DNA methylation, histone modification and micro RNA level, thus to decrease generation, and development of cancer.^[4]^ According to previous meta-analysis and epidemiologic studies, tea consumption was associated with decreased risk of prostate,^[5]^ gastric,^[6]^ biliary tract,^[7]^ breast,^[8]^ ovarian,^[9]^ lung,^[10]^ and liver^[11]^ cancers, but has no significant link of pancreatic^[12]^ and bladder^[13]^ cancer.

Galeone et al have summarized data from 9 studies which were included in the International Head and Neck Cancer Epidemiology Consortium (INHANCE) and indicated that tea intake was not associated with oral cancer.^[14]^ However, 2 studies using meta-analysis method have suggested that tea consumption was associated decreased risk of oral cancer.^[15,16]^ The difference of above conclusions promoted us to perform a comprehensive and dose-response meta-analysis to clarify the relationship between oral cancer risk and tea consumption.

## 2 Materials and methods

### 2.1 Literature search

We performed an electric search up to Jul 21, 2018 by 2 independent researchers (HZ and WW) within Pubmed, Embase and Web of Science databases, using the following terms: “tea”, “black tea”, “green tea”, “oolong tea”, “oral”, “oral cavity”, “cancer”, “tumor”, “carcinoma”, “neoplasm,” and “malignance”. The reference lists from relevant original studies were manually reviewed for selecting potential omitted studies. No language restrictions were imposed in this meta-analysis.

### 2.2 Study selection

The eligible inclusion criteria were as followed:

(i) the study design was a case-control;
(ii) studies reported the associations of histological diagnosed oral cancer risk with tea intake;
(iii) odds ratio (OR) with 95% confidence interval (CI) or detailed original data was given;
(iv) the number of cases and controls and eligible dose (mL/day or cups/day), consisting time (year) or concentration of tea must be provided for dose-response analysis.

Other units of measurement were excluded for limited number of studies. The exclusive criteria included:

(i) inadequate data to evaluate the association between oral cancer and tea consumption;
(ii) study evaluating the cancer risk from oral cavity and pharyngeal with tea intake. The most comprehensive study was retrieved for duplicate publications.

### 2.3 Data extraction and quality assessment

Two researchers extracted relevant data from the included studies independently using a standardized manner. The following information was collected: publication year, the first author's name, people source, study design, gender, sample size, age, dose of tea, types of tea, concentration of tea, duration time (year) of tea intake, control source, population source, adjusted variables, risk estimates, and 95% CI for evaluating the risk of oral cancer and tea consumption. We selected the maximally adjusted ration as the only evaluation index for preventing possible confounders when studies reported multivariable adjusted-effect estimates. Once the study did not report the data evaluate the overall relationship between tea intake and oral cancer risk, the results were pooled first before being used in the final analysis. The number of case and total participate for each category of tea consumption was also included for dose-response analysis. All controversial questions were resolved by asking a third author.

Newcastle–Ottawa Quality Assessment Scale (NOS), which has been validated as an available tool for assessing the quality of observational studies in meta-analysis, were chosen to evaluate the quality of included studies.^[17,18]^ The details of NOS score were as followed including total 9 sub-classifications (1 score/item): selection of participants and measurement of exposure (4 items), comparability (2 items), and evaluation of methodological quality outcome (3 items). Study evaluated with 7 score or more was considered as high level of research.^[19,20]^

### 2.4 Statistical analysis

Statistical heterogeneity was assessed by the I^2^ statistic and χ^2^ test. For the I^2^ metric, the cut-off points were set as 25%, 50%, and 75% for low, moderate and high degrees of heterogeneity, respectively. When heterogeneity was significant (*P* ≤.1 or I^2^ ≥ 50), the random effect model was chosen, otherwise, the fixed effect model was chosen. Pooled risk estimates (OR) with 95% CI were used for evaluating the links of tea consumption with risk of oral cancer.^[21,22]^ Sensitivity test was conducted to assess robust of pooled results by removing 1 literature each time when the heterogeneity was significant. The publication bias was conducted by the Egger test, Begg test and a macroscopic vision on funnel plots.^[23]^*P* <.05 was considered as statistically significant and existing potential publication bias, and all *P* values were 2-sided.

Stratified analyses were performed by population, gender, age, type of tea, control source, duration of tea intake, study quality (NOS scores), and population source.

To enable the dose-response analysis, the number of cases and total participates and ORs with 95% CI estimating for at least 3 quantitative exposure categories from each study were extracted. If studies did not provide tea intake in terms of cups, 120 mL/day or 50 g/day of tea consumption was considered equal to 1 cup/day roughly.^[24,25]^ The midpoint of tea intake in each category was set as the dose of tea consumed or duration time of tea intake. If the highest category was open-ended, the midpoint of the category was set as 1.5 times the lower boundary. If the lowest category was open-ended, we set the lowest boundary value at 0.^[26]^ We assessed the concentrations of tea intake as 4 grades: nondrinking, low level, moderate level, and high-level corresponding to the given values of 0, 1, 2, and 3, respectively. To drive the dose-response curve, we used the restricted cubic splines with 4 knots at the 5%, 35%, 65%, and 95% percentiles of the distribution to evaluate the potential curvilinear relations of tea intake and oral cancer risk.^[27,28]^ All statistical analysis was performed using Stata 12.0 (StataCorp LP, College Station, TX). The ethical statement is not necessary for this study.

## 3 Results

### 3.1 Summary of studies’ characteristics

**Figure 1:**
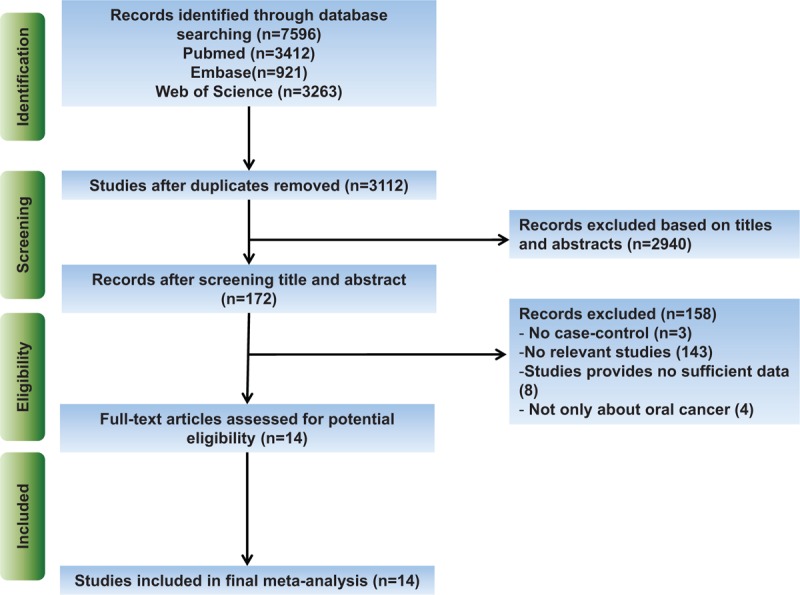
The flow diagram of the literature search, analysis, and selection of studies used in the current meta-analysis.

Figure 1 shows the eligible literature selecting process for the meta-analysis. Totally, 7596 potential articles were obtained from initial search. After automatic excluding duplicated studies within Endnote, 3112 studies were remained. After screening titles and abstracts, 2940 studies were excluded. Finally, according to our inclusive criteria mentioned in Materials and Methods, 14 articles were identified and included in the meta-analysis.^[29–42]^

The main characteristics of the included studies are listed in Table 1. Through eligible literature searching process, 14 case–control studies^[29–42]^ including 5920 patients and 10553 controls were selected. Eight of them were performed in Asia,^[29–33,35,38,41]^ 2 studies were performed in America,^[40,42]^ 3 studies were conducted in Europe,^[34,37,39]^ and 1 study in Africa.^[36]^ Three studies separately reported the mean age of participants <60 or >60 years.^[30–32]^ Eight studies used non-drinkers as control group,^[29–34,37,42]^ and 6 studies used lowest drinkers as control group.^[35,36,38–41]^ Four studies investigated the association between the tea intake and the risk of oral cancer in women,^[30–32,35]^ and 3 studies evaluated this association in men.^[30,31,35]^ Six studies detected the association between green tea and the risk of oral cancer,^[30–33,35,38]^ 3 studies assessed the association about black tea,^[31,35,38]^ and 4 studies assessed the association about oolong tea.^[30–33]^ Seven articles matched the high criteria of NOS,^[30–34,37,38]^ and 7 researches gained low scores.^[29,35,36,39–42]^

**Table 1:**
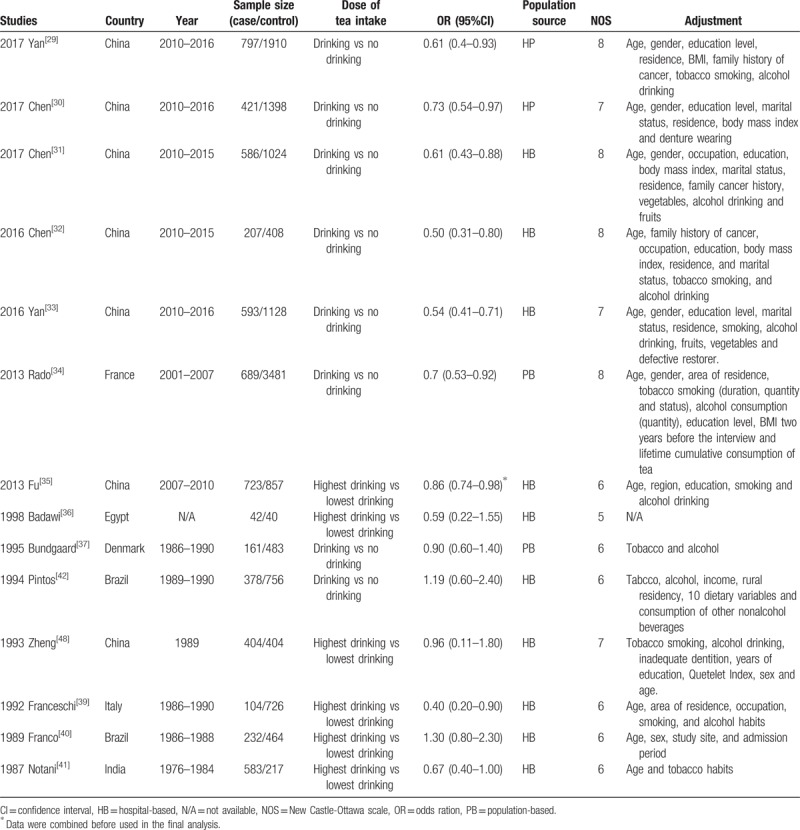
Characteristics of studies included in the current meta-analysis evaluating associations between tea and risk of oral cancer.

### 3.2 Overall meta-analysis

**Figure 2:**
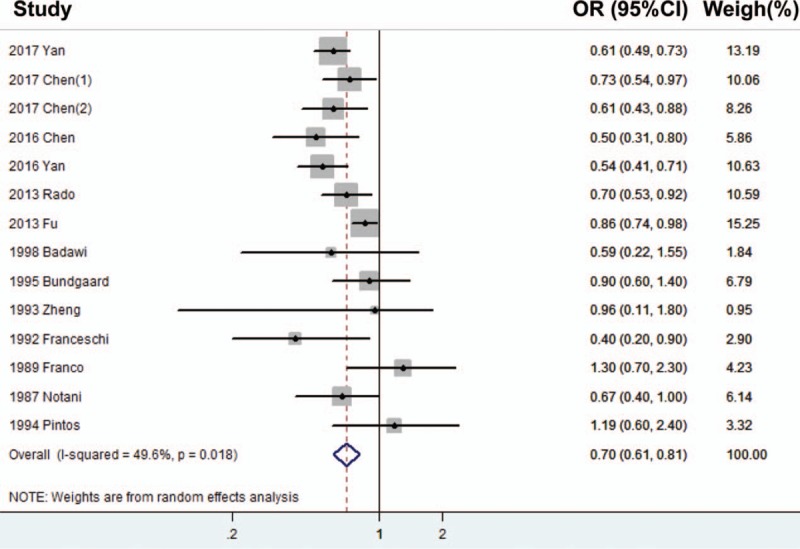
Forrest plot showing the relationship between tea intake and the risk of oral cancer.

Totally, 14 case–control studies containing 5920 patients and 10553 controls were included to evaluate the association between tea consumption and oral cancer risk. The random effect model was selected for the significant heterogeneity (*P* = .018, *I*^2^ = 49.6%). The pooled OR was 0.700 (95% CI = 0.609–0.805, *P* <.001, Table 2, Fig. 2), which suggested an inverse association between tea consumption and risk of oral cancer.

**Table 2:**
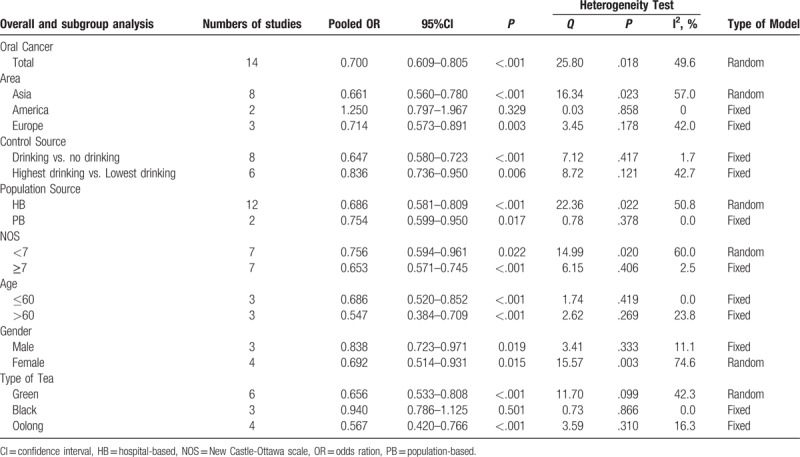
Comprehensive and stratified analysis of the relationships between risk of oral cancer and tea consumption.

### 3.3 Subgroup analysis

Detailed stratified analysis was performed according to the categories of area, control source, population source, NOS, age, gender, and type of tea. Except for subgroup analysis of American people (OR = 1.250, 95% CI = 0.797–1.967, *P* = .329, Fixed-effect model, Table 2) or black tea (OR = 0.940, 95% CI = 0.786–1.125, *P* = .501, Fixed-effect model, Table 2) suggesting that tea consumption was not associated with the risk of oral cancer, other results showed significant inverse relationships (*P* <.05) between tea consumption and the risk of oral cancer.

### 3.4 Dose-response analysis

**Figure 3:**
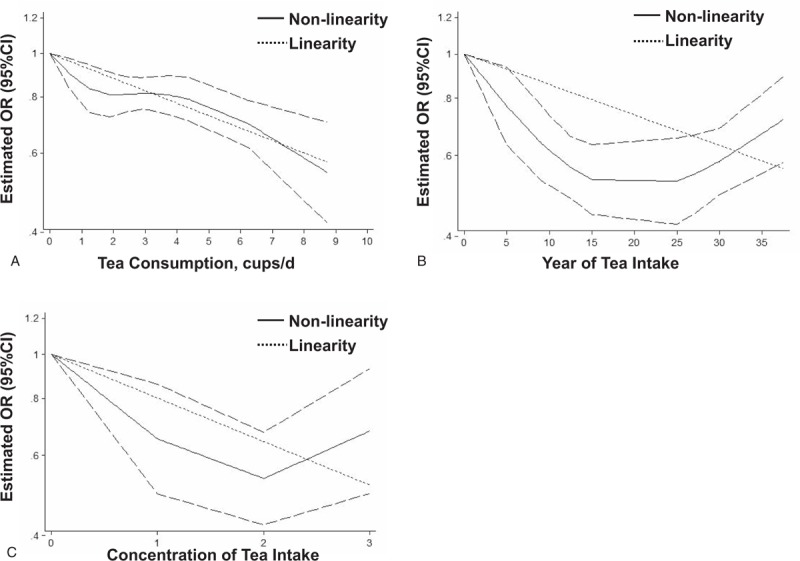
Linear and non-linear dose–response relationships between the risk of oral cancer and dose (A), duration time (B) and concentration (C) of tea intake. For concentration of tea intake, 0, 1, 2, and 3 corresponding to nondrinking, low level, moderate level and high level, respectively.

To determine the dose relationship between tea consumption and oral cancer risk, a dose-response analysis was performed. Eight case–control^[29–35,40]^ were collected to assess the dose relationship between dietary tea consumption and the risk of oral cancer. As shown in Figure 3A, the lowest OR was 0.54 (0.42–0.70) at the highest dose of 8.75 cups/day intake. The linearity test of dose-response analysis suggested that with increased 1 cup/day tea consumption, the risk of oral cancer decreased 6.2% degree (OR = 0.938, 95% CI = 0.922–0.955, *P* <.001) at an appropriate dose range of 0 to 8.75 cups/day. The non-linearity test also indicated an inverse association between dietary tea intake and oral cancer risk (*P* <.001).

Six case–control studies^[29–34]^ were included in the dose-response analysis to evaluate the duration years of tea intake and the risk of oral cancer. As shown in Figure 3B, the linearity test of dose-response analysis suggested that with increased 5 years of tea consumption, the risk of oral cancer reduced 7.4% (OR = 0.926, 95% CI = 0.905–0.948, *P* <.001). There was a significant non-linear dose-response relationship between the duration years of tea intake and the decrease in the risk of oral cancer (*P* <.001).

Four case-control studies^[30–33]^ were included in the dose-response analysis to evaluate the concentration of tea intake and the risk of oral cancer. As shown in Figure 3C, the linearity (OR = 0.802, 95% CI = 0.740–0.870, *P* <.001) and non-linearity (*P* <.001) tests all suggested that with the concentration of tea consumption increased, the risk of oral cancer decreased.

### 3.5 Publication bias and sensitivity analysis

**Figure 4:**
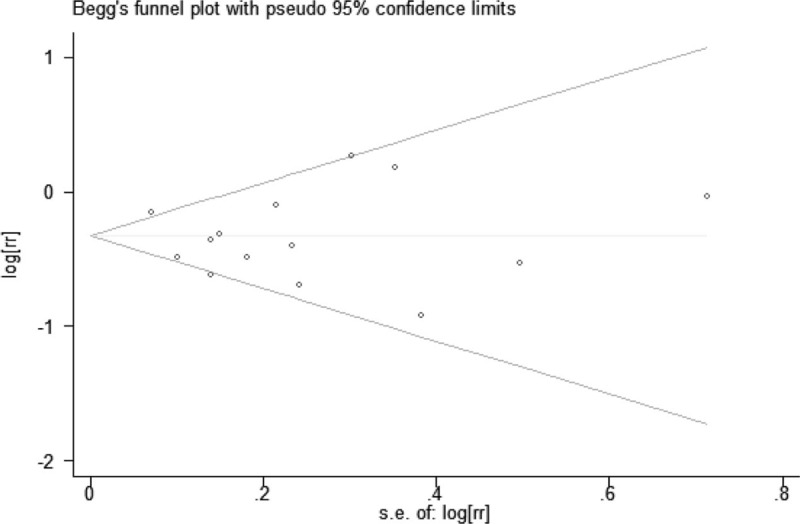
Begg's funnel plot for studies included in the meta-analysis of the relationship between tea intake and oral risk.

Begg test and Egger test were used to evaluate the potential publication bias of included studies. The Begg test (*P* = .743) and Egger test (*P* = .635) all were not significant which suggested no significant publication was found in this meta-analysis. In addition, funnel plots did not show substantial asymmetry (Fig. 4).

**Figure 5:**
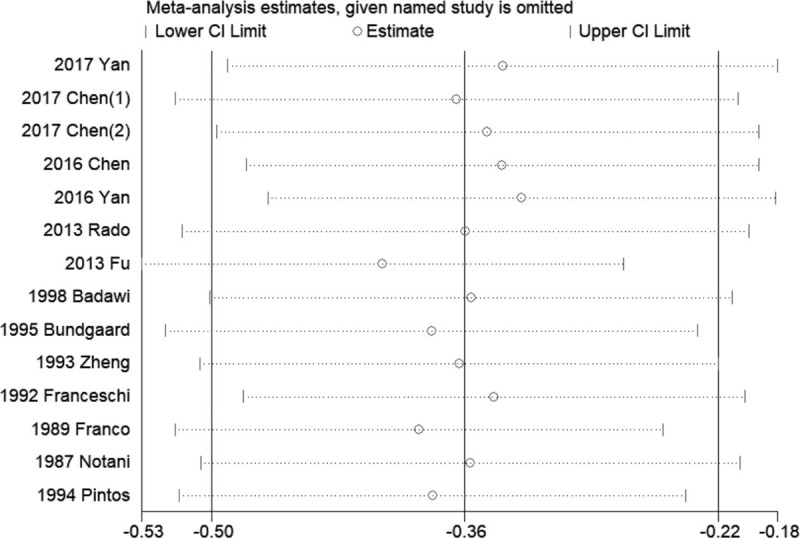
Sensitivity analysis of pooled ORs in this meta-analysis. OR = odds ration.

The comprehensive (Fig. 5) and subgroup analysis of sensitivity analysis were performed by omitting 1 study each time and showed robust and stable results of pooled data in this meta-analysis.

## 4 Discussion

Our results of this meta-analysis suggested that increased tea consumption was associated with a decreased risk of oral cancer. In addition, dose-response analysis indicated that the risk of oral cancer reduced continuously with dose, duration time, and concentration of tea consumption increasing. Moreover, the subgroup analysis of American people and black tea indicated that tea intake was not associated with the risk of oral cancer.

Tea is a commonly consumed beverage worldwide and more popular than coffee, wine and carbonated soft drinks.^[4]^ Polyphenols, the main bioactive molecules in tea, are widely found in various teas. Catechins are considered as the major polyphenolic compounds in tea.^[4]^ Among different catechins, epigallocatechin-3-gallate (EGCG) is the most prevalent and believed as the main molecule to prevent carcinogenesis. Several mechanisms are accepted to explain this anti-cancer effect of EGCG. First, EGCG is found to change the activity of DNA methyltransferase enzymes and reactivate methylation-silenced genes in different cancer cells in vivo and in vitro.^[43–46]^ Second, it was reported that EGCG decreased expression of Class I histone deacetylases, which increased global acetylation of histone proteins and suppressed cancer growth.^[47,48]^ Third, EGCG was certificated to induce cancer cell apoptosis by regulating several miRNAs expression.^[49,50]^ However, the specific mechanism that tea prevents formation of oral cancer still remains unclear.

According to the original data from INHANCE, Galeone and his colleagues reported that tea intake was not associated with the risk of oral cancer.^[14]^ However, this opinion was opposed by 2 published meta-analysis which considered that tea consumption was associated with decreased risk of oral cancer.^[15,16]^ In the present study, our results suggested that tea consumption was associated with decreased risk of oral cancer. Importantly, the dose-response analysis also certificated the inverse relationship and indicated that with every 1 cup of dietary tea intake increasing, the oral cancer risk decreased 6.2% degree by a linearity test. To strength, the results, detailed subgroup analysis was performed. Interestingly, our results showed that increased tea consumption was not associated with oral cancer risk in American which suggested that ethnics and gene polymorphism may have a significant affection on the effect of tea consumption in oral cancer prevention. In addition, stratified analysis based on type of tea indicated that black tea consumption was not associated with oral cancer risk. One possible reason is that most polyphenols of black tea are dimerized due to extended oxidation with enzymatic reactions and deactivated effect of anti-cancer during the process of preparation.

Our study has several strengths. First, it is the first dose-response meta-analysis to evaluate the linearity and non-linearity relationships at different exposure levels of tea consumption and oral cancer risk. Second, we analyzed not only the relationship between drinking tea dose and the risk of oral cancer but also the links between duration time and concentration of tea intake and risk of oral cancer by the dose-response method. Finally, detailed subgroup analysis was performed to investigate the origin of heterogeneity, which made our results more reliable.

However, some limitations were inevitable in our study. First, for lack of associated cohort studies, we only included case–control studies, which may lead some publication bias and selection bias; Second, we used pooled data (individual data were not provided) as the final analysis in our meta-analysis, which prevented the further exact analysis and precise results from being obtained. Third, although no language restriction was found in the present meta-analysis, some studies may be missed because the database searches were limited. Finally, in the dose-response analysis, we have transformed 120 mL/d to 1 cup/d according to previous publication suggestions. However, this transformation was crude and may result in the inaccuracy of the last conclusion. Hence, our results should be interpreted with caution.

In conclusion, the results of our meta-analysis indicated an inverse association between the tea intake and the risk of oral cancer. Dose-response analysis suggested that dietary high dose, long-term and high concentration of tea intake may be associated with the reduced risk of oral cancer. Finally, our study suggested more large-scale pooling and high-quality prospective studies are necessary for detecting the precise relationship between tea intake and oral cancer risk in the future.

## Author contributions

ZC designed the study. HZ and WW performed the search. WW and FW prepared the tables and figures. ZC and HZ wrote the manuscript. All author reviewed the manuscript.

**Data curation:** Fengqin Wang.

**Formal analysis:** Fengqin Wang.

**Investigation:** Hao Zhou, Weiwei Wu.

**Software:** Weiwei Wu.

**Writing – original draft:** Zhigang Cheng.

**Writing – review & editing:** Huizhong Qi, Zhigang Cheng.
